# Acute Kidney Injury Following Transcatheter Aortic Valve Implantation: Association with Contrast Media Dosage and Contrast Media Based Risk Predication Models

**DOI:** 10.3390/jcm11051181

**Published:** 2022-02-23

**Authors:** Doron Sudarsky, Yarden Drutin, Fabio Kusniec, Liza Grosman-Rimon, Ala Lubovich, Wadia Kinany, Evgeni Hazanov, Michael Gelbstein, Edo Y. Birati, Ibrahim Marai

**Affiliations:** 1 The Department of Cardiology, The Lidya and Carol Kittner, Lea and Benjamin Davidai Division of Cardiovascular Medicine and Surgery, B. Padeh Medical Center, Poriya 1528001, Israel; fkusniec@poria.health.gov.il (F.K.); l.grosman.rimon@gmail.com (L.G.-R.); alubovich@poria.health.gov.il (A.L.); wkinany@poria.health.gov.il (W.K.); ehazanov@poria.health.gov.il (E.H.); mgelbstein@poria.health.gov.il (M.G.); ebirati@poria.health.gov.il (E.Y.B.); imarai@poria.health.gov.il (I.M.); 2 The Azrieli Faculty of Medicine in the Galilee, Bar-Ilan University, Safed 1311502, Israel; yardi.wohl@gmail.com

**Keywords:** transcatheter aortic valve implantation (TAVI), acute kidney injury (AKI), contrast media (CM), risk models

## Abstract

The effect of contrast media (CM), delivered prior to- and during transcatheter aortic valve implantation (TAVI), on kidney function, following the procedure, is debatable. Consequently, the performance of CM-based, acute kidney injury (AKI) risk prediction models is also questionable. We retrospectively studied 210 patients that underwent TAVI. We recorded the dose of CM used prior and during TAVI, calculated the results of different AKI risk assessment models containing a CM module, and tested their association with AKI after the procedure. AKI was diagnosed in 38 patients (18.1%). The baseline estimated glomerular filtration rate (eGFR) was lower in the AKI+ group compared to AKI− group (51 ± 19.3 versus 64.5 ± 19 mL/min/1.73 mr^2^, respectively). While the dose of CM delivered prior to TAVI, during TAVI or the cumulative amount of both did not differ between the groups, the results of all tested risk models were higher in AKI+ patients. However, by multivariable analysis, only eGFR had a consistent independent association with AKI. We suggest that the dose of CM delivered prior or during TAVI is not associated with AKI and that the predictive power of CM based AKI risk models is, in all probability, limited to eGFR alone.

## 1. Introduction

Acute kidney injury (AKI) following transcatheter aortic valve implantation (TAVI) is frequent and strongly associated with adverse outcomes [1,2,3,4,5]. Therefore, it is imperative to recognize the parameters that inflict an increased risk for AKI following TAVI. Previous studies have linked, although inconsistently, the volume of radiographic contrast media (CM) administered during TAVI, with the development of AKI after the procedure [5,6,7,8,9,10]. It is possible, although not tested thus far, that preparatory exposure to CM, before TAVI, may impose additional deleterious effects on post-TAVI kidney function. Risk assessment tools, incorporating CM volume, have proved useful in predicting the development of AKI following percutaneous coronary procedures [11,12,13,14]. However, the utility of such risk models in predicting post-TAVI AKI is less established [6,15,16].

We investigated the association between the total volume of CM delivered during TAVI as well as during near-past CM utilizing procedures and the occurrence of post-TAVI AKI. We additionally tested the ability of CM based AKI risk assessment models, previously verified with percutaneous coronary interventions (PCI), to predict post-TAVI AKI. We also examined whether including previous exposures to CM, before TAVI, would improve the accuracy of those risk models in predicting AKI after TAVI.

## 2. Materials and Methods

We conducted a retrospective analysis using patient data from Poriya Medical Center’s (PMC) local TAVI database. The study complied with the ethical guidelines of the Declaration of Helsinki, was approved by the Ethical Review Board at PMC, and each patient provided written informed consent before the intervention. Data were recorded prospectively during the index hospitalization as well as during follow-up visits. Patients were excluded from the analysis if they had one or more of the following: (1) treatment with renal replacement therapy prior to TAVI, (2) missing data about the volume of CM prior or during TAVI, (3) a death within seven days of TAVI without being diagnosed with AKI, (4) missing follow-up AKI data, or (5) additional exposures to CM between one day and seven days after the completion of TAVI. 

We grouped and compared the patients according to the diagnosis of AKI (AKI+), following TAVI, or freedom from AKI (AKI-). AKI was defined according to the Valve Academic Research Consortium-3 standardized endpoint definitions as follows: an increase in serum creatinine (SCr) ≥ 150–200% within seven days compared with baseline or increase of ≥0.3 mg/dL within 48 h of the index procedure was listed as AKI stage-1. An increase in SCr > 200–300% within seven days compared with baseline was recorded as AKI stage-2, an increase in SCr > 300% within seven days compared with baseline or SCr ≥ 4.0 mg/dL with an acute increase of ≥0.5 mg/dL was indexed as AKI stage-3, and AKI requiring new temporary or permanent renal replacement therapy was indexed as AKI stage-4 [17]. AKI+ depicts AKI stages 1 through 4, while AKI− represents the absence of AKI.

The following parameters were recorded: (1) baseline clinical and echocardiography data, (2) the volume of CM delivered during TAVI, and (3) the total volume of CM administered during the time intervals that started either seven days or 30 days before TAVI and ended at the completion of TAVI. We included all CM delivered during percutaneous diagnostic and interventional coronary and peripheral procedures as well as during computed tomography angiography (CTA) performed within the previously stated time intervals. In the cases where there were additional exposures to CM, within 24 h of TAVI, the volume of CM delivered during those exposures was added to the volume of CM administered during TAVI.

We used Omnipaque™ 350 (GE Healthcare, Cork, Ireland) in all procedures. Omnipaque™ 350 is a low-osmolar, non-ionic, water-soluble, radiographic CM containing 755 mg/mL of Iohexol equivalent to 350 mg/mL of organic iodine (Osmolality 844 mOsm/kg water, Osmolarity 541 mOsm/L, absolute viscosity at 37 °C 10.4 cp, specific gravity at 37 °C 1.406).

The estimated glomerular filtration rate (eGFR) was calculated with the Chronic Kidney Disease Epidemiology Collaboration (CKD-EPI) equation [18]. The CKD category was determined according to the Kidney Disease Improving Global Outcomes (KDIGO) criteria [19]. The risk of a post-procedural AKI was calculated using various, previously published risk models utilizing CM volume delivered during TAVI. We also calculated those risk models using the cumulative volume of CM administered during a seven-day or 30-day interval, as described above. Additionally, when possible, we calculated a modified version of the risk model in which the CM volume was excluded. The following AKI risk prediction models were tested: (1) CM volume (in ml) divided by the creatinine clearance (CrCl) (in mL/min/1.73 m^2^) [11], (2) CM (in ml) × Serum Creatinine (SCr) (in mg/dL)/Body weight (BW) (in kg) [10], (3) CM (in ml) × SCr (in mg/dL)/body mass index (BMI) (in kg/m^2^) [16], (4) The Mehran risk score was calculated by adding the following: congestive heart failure, 5 points; hypotension, 5 points; intra-aortic balloon pump use, 5 points; age over 75 years, 4 points; anemia, 3 points; diabetes mellitus, 3 points; CM volume, 1 point per 100 mL; eGFR, 2 points for 40–59 mL/min/1.73 m^2^, 4 points for 20–39 mL/min/1.73 m^2^ and 6 points for <20 mL/min/1.73 m^2^) [13], and (5). The CR4EATME3AD3 model was calculated as follows: contrast volume > 200 mL, 2 points; eGFR < 60 mL/min/1.73 m^2^, 4 points; emergency procedure, 2 points; age > 70 years, 2 points; hypotension, 2 points; previous myocardial infarction, 2 points; left ventricular ejection fraction < 45%, 3 points; anemia, 2 points; diabetes mellitus, 3 points [14].

Statistical analysis: Categorical variables were reported as absolute numbers and percentages and were compared using the chi-square test. Continuous data with normal distribution were reported as the mean and standard deviation (SD) and were compared using an independent samples *t*-test. Continuous variables with a non-normal distribution were reported as medians and interquartile ranges (IQR), and were compared using the Mann–Whitney U test. Univariable and multivariable logistic regression analyses were performed to evaluate for predictors of AKI following TAVI with reported odds ratios (OR’s) and 95% confidence intervals (CI). We excluded any variable with more than 10% missing values. First, univariable associations were determined for all potential covariates. Covariates with a *p* value of less than 0.1 on univariate analyses were entered into the multivariable logistic regression models. Risk variables that were determined to be significant in the univariable analysis were subsequently tested with the multivariable modeling. A 2-sided *p*-value of ≤0.05 was considered statistically significant.

## 3. Results

**Figure 1:**
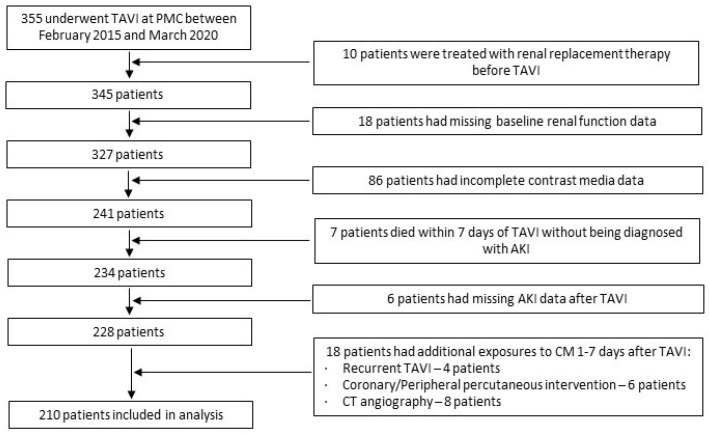
Patient selection flow chart.

Between February 2015 and March 2020, a total of 355 patients underwent TAVI at PMC. One hundred and forty-five patients were excluded from the analysis. The reasons for excluding these patients are depicted in Figure 1.

The baseline data of the remaining 210 patients, which were included in the analysis, is summarized in Table 1. AKI was diagnosed in 38 patients (18.1%). Of them, 26 (68.4%) patients were classified as suffering from stage-1 AKI, eight (21.1%) patients were classified as stage-2 AKI, three (7.9%) patients had stage-3 AKI and one (2.6%) patient was diagnosed with stage-4 AKI. For the most part, the two groups had a similar baseline profile with the exception of a few significant differences. AKI+ patients, compared to AKI− patients, had a higher prevalence of prior neurological insult, higher surgical risk, as assessed by the STS score, lower hemoglobin levels and a higher rate of anemia (86.8% versus 69.2%, respectively, *p* = 0.028). They also had worse baseline kidney function with higher baseline creatinine and lower eGFR. Overall, 60.5% of AKI+ patients had eGFR ≤ 60 mL/min/1.73 m^2^ compared to 37.2% of AKI− patients (*p* = 0.008). Baseline echocardiographic data was not found to be different between the two groups. 

After TAVI, AKI+ patients, compared to their counterparts, had higher SCr and lower eGFR during their post procedural hospitalization both at short-term (i.e., 30 days) and long-term (i.e., 12-month) follow-up (Table 2).

CM data and risk models data, at the different tested time intervals, indexed according to the presence or absence of AKI after TAVI, are presented in Table 3. AKI+ patients and AKI− patients did not significantly differ in the volume of CM administered during TAVI. Nor did they differ in the amount of CM delivered, prior to TAVI, within a seven-day or a 30-day periods or in the cumulative amount of CM administered during all procedures. The scores for all tested risk models were significantly higher for AKI+ patients compared to AKI− patients either when they included the volume of CM administered during TAVI or the cumulative volume of CM as described earlier. Additionally, the modifications of Mehran risk model and CR4EATME3AD3 model, which were calculated without the CM element, were also higher in AKI+ patients.

Table 4 shows the results of univariable analysis. Baseline eGFR < 45 mL/min/1.73 m^2^, hemoglobin level and the STS score were significantly associated with AKI following TAVI. Additionally, all risk models, whether computed without the CM volume module or with it, as administered during TAVI or during the prespecified seven-day and 30-day intervals, were also predictive of AKI. In contrast, neither the administered volume of CM during TAVI nor the seven-day or 30-day cumulative dose of CM were associated with AKI development.

Each of the risk models was discretely entered into a multivariable logistic regression model together with the hemoglobin level, eGFR < 45 mL/min/1.73 m^2^ and STS score. By multivariable analysis none of the risk models predicted post-TAVI AKI. Neither did the hemoglobin level nor the STS score. Only pre-TAVI eGFR, in most of the analyses, was independently associated with AKI after it (Table 5).

## 4. Discussion

The main findings of the study were: (1) The volume of CM administered during TAVI and the cumulative volume of CM administered within seven days or within 30 days from TAVI were not associated with AKI post-TAVI; and (2) None of the tested risk assessment models that included a CM module, were independently associated with post-TAVI AKI. (3) eGFR was the only independent predictor for AKI. 

AKI is a serious complication, commonly encountered following TAVI with an incidence of any level of AKI, reported from large registries and metanalyses, at around 20% [1,3]. The development of AKI, following TAVI, significantly increases the risk of both short-term and long-term deleterious complications, such as myocardial infarction, life threatening bleeding, permanent renal dysfunction necessitating renal replacement therapy, and mortality [1,3,4,5,20,21,22,23]. Various baseline characteristics, co-morbidities and peri-procedural factors have been linked with TAVI related AKI. Some of the most reported are the baseline decreasing level of hemoglobin, chronic kidney dysfunction, acute bleeding necessitating blood transfusions and hemodynamic instability during the procedure [2,5,22,24,25]. Still, there is much uncertainty with a multitude of proposed risk factors previously associated with AKI following other procedures in which CM is used, and the role they play in the development of AKI following TAVI with conflicting reported results. For instance, while some studies demonstrated a relationship between increasing age and AKI development following TAVI, other studies did not [3,6,7,10,25]. While in some studies LVEF was dis-concordantly associated with post-TAVI AKI, in other publications it was not [6,7,10,23,25,26]. Even decreasing baseline eGFR and chronic kidney dysfunction have an uncertain magnitude of effects on post-TAVI AKI [3,6,7,8,10,25,26,27]. The relationship between CM volume and TAVI-related AKI is also controversial. In several previous studies there was no significant difference in the volume of CM delivered during TAVI, between AKI+ patients and AKI− patients, nor between CM dose and post-procedural AKI [2,4,5,8,16]. Conversely, other studies described higher dosages of CM in AKI+ patients compared to AKI, and an association between the volume of CM delivered during TAVI and the subsequent development of AKI [6,9,10,25,26].

The uncertain association between administered CM volume and AKI has been described with other medical procedures utilizing radiographic CM. In patients with eGFR above 30 mL/min/1.73 m^2^ who underwent CT testing, there was no significant difference in the incidence of AKI between those who received intravenous CM and those who did not, and the results for patients with eGFR less than 30 mL/min/1.73 m^2^ were conflicting [28,29]. An analysis of observational studies concluded that radiocontrast use in CT scanning was not causally related to changes in kidney function [30]. These results were echoed in the most recent update of the American College of Radiology consensus statement in which it was declared that the risk of AKI developing following exposure to intravenous iodinated CM has been exaggerated and that the true risk of AKI, related to CM administration, remains uncertain even for patients with severe kidney disease [31]. It has been suggested that intravenous administration of CM, like with CT angiography, may impose a different risk of AKI than the one associated with arterial administration of CM, like with PCI and TAVI; however, the data is inconclusive [32,33,34,35,36,37]. 

CM related AKI has been extensively studied in the context of PCI. However, assessing the true impact of CM on the development of AKI, following PCI, has proven to be a difficult task. Studies that evaluated this coupling between CM and AKI widely differed in the type, chemical characteristics, pharmacokinetics and volume of CM, the route it was administered, the medical procedure it was given for and in the definition used to diagnose AKI [38]. Notably, a broad range of contrast volume (from below 100 mL to above 800 mL) has been associated with post-PCI nephropathy [12,13,39,40,41]. There are several fundamental differences between patients treated with TAVI and patients treated with PCI which, potentially, position the former at higher risk to develop AKI than the later. Patients undergoing TAVI are usually elderly, fragile, frequently suffering from multiple co-morbidities and have reduced GFR [7,42]. Additionally, PCI-treated patients are usually exposed to CM once during their index procedure. TAVI treated patients, however, are frequently exposed to a large cumulative volume of CM during several diagnostic and interventional procedures and over a relatively short period of time. Interestingly, despite these differences, Venturi et al. found that AKI actually occurred less frequently in patients undergoing TAVI than in patients undergoing PCI, even after propensity score matching [7]. Following TAVI, unlike other procedures that utilize CM, there is an immediate and sustained improvement in hemodynamics resulting in an increase in cardiac output and systemic perfusion [43,44,45]. Still, even though this can possibly translate into the reduced incidence of AKI following TAVI, the rate of immediate kidney function improvement after TAVI was found to be small in a recent publication (5%) [46]. 

The diagnosis of AKI tends to lag after the index exposure to CM, with gradual worsening of kidney function over the ensuing days. Accordingly, the assessment for CM associated nephrotoxicity was traditionally made within 48 to 72 h of exposure and according to VARC-3 consensus statements this time frame has been further extended to seven days [13,17,39]. Thus far, the effect of CM on kidney function was tested for CM delivered during TAVI alone, while the possible effect inflicted by near past exposures to CM was overlooked. This led us to test for an additive effect of preceding exposures to CM on the incidence of AKI following TAVI. In this study, the volume of CM administered during TAVI alone, during a 7-day period or during a 30-day period, did not significantly differ between AKI+ patients and AKI− patients, nor did it independently predict the development of AKI. Similarly, in a recent study, assessing the effect of multiple exposures to CM during recurrent diagnostic and interventional coronary procedures, although over a period of several years, worsening of renal function was associated with known risk factors for the progression of kidney disease but not with cumulative CM volume [47].

The difficulties in predicting AKI following TAVI led to the development of risk assessment models. Incorporating several parameters to form a risk model, can potentially improve the predictive power beyond that of the individual modules included in it. AKI risk prediction models have been developed, tested and validated for coronary procedures [11,12,13,14,48,49]. Sadly, only a few of these models have been specifically designed to assess for the risk of post-TAVI AKI. For instance, Zivcovic et al. introduced an AKI risk calculator that was meant to be utilized prior to TAVI and therefore did not include a CM component [27]. As in this study, several previous studies tested the effectiveness of CM-based, non-TAVI dedicated risk models in predicting AKI following TAVI [6,15,16,24,26,50]. In our study, none of the CM-based risk models we tested independently predicted post-TAVI AKI. Mach et al. reported that none of the six tested CM-based risk models (including the Mehran risk model) were significantly different between AKI+ and AKI− patients or, similarly to our results, independently predicted AKI [16]. Rosa et al. describes that all of their tested risk models, of which four included a CM module and two did not, had poor accuracy in terms of predicting the occurrence of any AKI. However, there was an improvement in AKI risk prediction for more advanced stages of AKI. By univariable logistic regression analysis, only risk models with a CM module were associated with AKI. The results of multivariable logistic regression coefficients of risk for any AKI or the different stages of AKI were not reported [15]. In contrast, a small study of 93 patients, of which AKI was diagnosed in 24 of them, found in a univariable analysis that the Mehran risk model, as well as CM volume, predicted post-TAVI AKI. The authors also reported that in multivariable analysis, CM volume, the Mehran score, SCr and eGFR were all independently associated with AKI [6]. However, the study was of limited sample size and, given the low number of endpoints achieved, was underpowered to accurately explore the aforementioned endpoints. 

In our study, decreasing eGFR, as previously described by others, was significantly and independently associated with AKI across most analyses [5,8]. In fact, it was the only independent predictor for AKI. Therefore, with caution, we suggest that the AKI predictive power of risk score models containing a CM volume module, is almost entirely limited to pre-procedural eGFR. 

Limitations: This is an individual center report and has the limitations associated with retrospective analysis. Given the relatively small number of AKI events, our study was underpowered to evaluate for the most severe stages of AKI. The assessment of baseline sCr was made prior to TAVI and not prior to the first exposure to CM within the assessed time interval. Additionally, most patients were discharged before seven days had elapsed from their procedure and therefore, even though it is our institutional practice to discharge the patient after kidney function has stabilized, it is possible that further deterioration in kidney function ensued after discharge without being recorded. The use of drugs with nephrotoxic properties close to contrast media exposure was also not recorded. 

## 5. Conclusions

Neither the volume of CM delivered during TAVI nor the cumulative amount of CM delivered during a time period that starts either seven-days or 30 days before TAVI and ends with TAVI are associated with AKI. The power of non-TAVI dedicated CM based risk models, in predicting AKI, is limited to pre-procedural kidney dysfunction.

## Figures and Tables

**Table 1:** Baseline characteristics.

			AKI (−) (*n* = 172)	AKI (+) (*n* = 38)	*p* Value
**Clinical data**	Age; Years		79 ± 7.9	81.8 ± 8.2	0.1
	Female; *n* (%)		97 (56.4)	21 (55.3)	0.9
	Body mass index; (kg/m^2^)		28.92 ± 5.17	28.2 ± 5.97	0.46
	Hypertension; *n* (%)		151 (87.8)	34 (89.5)	0.77
	Dyslipidemia; *n* (%)		133 (77.3)	23 (60.5)	0.032
	Diabetes mellitus; *n* (%)		76 (44.2)	20 (52.6)	0.34
	Smoking history; *n* (%)		26 (15.1)	6 (15.8)	0.92
	Cardio-vascular disease; *n* (%)		96 (55.8)	23 (60.5)	0.596
	Coronary artery disease; *n* (%)		91 (52.9)	23 (60.5)	0.728
	Peripheral arterial disease; *n* (%)		19 (11)	7 (18.4)	0.211
	Past PCI; *n* (%)		70 (40.4)	15 (41.7)	0.98
	Past CABG; *n* (%)		20 (11.6)	3 (7.9)	0.51
	Past CVA/TIA; *n* (%)		12 (7)	7 (18.4)	0.026
	Chronic obstructive pulmonary disease; *n* (%)		22 (12.8)	8 (21.1)	0.19
	Atrial fibrillation/atrial flutter; *n* (%)		43 (25)	15 (39.5)	0.071
	Pacemaker/CRT/ICD; *n* (%)		23 (13.4)	4 (10.5)	0.64
	NYHA functional class; *n* (%)	1	5 (2.9)	1 (2.6)	0.727
		2	133 (77.3)	29 (76.3)	
		≥3	8 (4.7)	3 (7.9)	
	Urgent TAVI; *n* (%)		40 (23.4)	10 (26.3)	0.7
	Surgical risk	STS, mortality; %	3.79 ± 2.37	4.91 ± 2.27	0.009
		EuroScore II; %	3.83 ± 3.71	4.7 ± 3.48	0.22
**Laboratory data**	Hemoglobin; g/dL		11.55 ± 1.71	10.77 ± 1.4	0.01
	Creatinine; mg/dL		1.07 ± 0.48	1.35 ± 0.58	0.002
	eGFR; mL/min/1.73 m^2^		64.5 ± 19	51 ± 19.3	<0.001
	CKD category; *n* (%)	1–2	109 (63.4)	15 (39.5)	<0.001
		3a–3b	54 (31.4)	17 (44.7)	
		≥4	9 (5.2)	6 (15.8)	
**Echo-** **cardiography** **data**	Left ventricle ejection fraction; %		58 ± 10.5	58 ± 11.1	0.917
	Aortic valve area; cm^2,^		0.77 ± 0.16	0.82 ± 0.19	0.166
	Aortic valve area index; cm^2^/m^2^		0.43 ± 0.07	0.46 ± 0.06	0.081
	Aortic valve mean pressure gradient; mmHg		43 ± 12	41 ± 14.8	0.58
	Severe mitral regurgitation; *n* (%)		11 (6.9)	3 (8.1)	0.737
	Severe tricuspid regurgitation; *n* (%)		6 (4)	1 (2.7)	0.79
	Severe pulmonary hypertension; *n* (%)		14 (9.5)	4 (11.4)	0.634
**Procedural data**	Trans-femoral access; *n* (%)		170 (98.8)	38 (100)	0.99
	THV type; *n* (%)	Evolut-R™ (Medtronic)	124 (72.5)	27 (71.1)	0.676
		SAPIEN-3™ (Edwards Lifesciences)	28 (16.4)	5 (13.2)	
		ACURATE-Neo™ (Boston Scientific)	19 (11.1)	6 (15.8)	
	Anesthesia Type; *n* (%)	General anesthesia	72 (51.4)	16 (48.5)	0.12
		Local anesthesia with sedation	68 (48.6)	17 (51.5)	

AKI—acute kidney injury; PCI—percutaneous coronary intervention; CABG—coronary artery bypass graft surgery; CVA—cerebrovascular accident; TIA—transient ischemic attack; CRT—cardiac resynchronization therapy; ICD—implantable cardioverter defibrillator; STS—society of thoracic surgeons; eGFR—estimated glomerular filtration rate; CKD—chronic kidney disease; THV—transcatheter heart valve.

**Table 2:** Post-TAVI renal function data.

		AKI (−) (*n* = 172)	AKI (+) (*n* = 38)	*p* Value
Creatinine; mg/dL	Highest (In-hospital)	1.08 ± 0.31	2 ± 1	<0.001
	30-day	1.12 ± 0.42	1.45 ± 0.55	0.011
	12-month	1.21 ± 0.5	2 ± 1	0.014
eGFR; mL/min/1.73 m^2^	Lowest (In-hospital)	62 ± 20	27 ± 12	<0.001
	30-day	60.9 ± 20.6	44.9 ± 19.2	0.005
	12-month	58.4 ± 21.3	44 ± 20.9	0.035

TAVI—transcatheter aortic valve implantation; AKI—acute kidney injury; eGFR—estimated glomerular filtration rate.

**Table 3:** Contrast media and risk models data.

	Within 30 Days			Within 7 Days			During TAVI		
	AKI (−) (*n* = 38)	AKI (+) (*n* = 172)	*p* Value	AKI (−) (*n* = 38)	AKI (+) (*n* = 172)	*p* Value	AKI (−) (*n* = 38)	AKI (+) (*n* = 172)	*p* Value
CM volume; mL	319 ± 92	320 ± 105	0.97	246 ± 89	259 ± 111	0.54	187 ± 53	201 ± 83	0.316
CM volume prior to TAVI; mL	133 ± 74	119 ± 65	0.41	59 ± 73	58 ± 71	0.93	NR	NR	NR
Mehran score	13.52 ± 4.15	16.07 ± 3.63	0.004	12.76 ± 4.06	15.46 ± 3.73	0.025	12.2 ± 4.05	14.72 ± 3.75	0.004
Modified Mehran score ^	NR	NR	NR	NR	NR	NR	10.33 ± 3.97	12.87 ± 3.87	0.004
CR4EATME3AD3	10.09 ± 3.88	12.03 ± 3.75	0.007	9.56 ± 3.87	11.55 ± 3.63	0.004	9.09 ± 3.87	11.13 ± 3.78	0.004
Modified CR4EATME3AD ^	NR	NR	NR	NR	NR	NR	8.45 ± 3.81	10.45 ± 3.85	0.006
(CM × SCr)/BMI	11.83 ± 6.27	15.52 ± 9.8	0.004	9.1 ± 5.25	12.36 ± 9.78	0.005	7.02 ± 4.29	9.33 ± 5.6	0.005
(CM × SCr)/BW	4.5 ± 2.41	5.85 ± 3.44	0.005	3.48 ± 2.04	4.69 ± 3.48	0.005	2.66 ± 1.61	3.58 ± 2.13	0.004
CM/CrCl	5.45 ± 2.66	7.04 ± 3.6	0.002	4.18 ± 2.25	5.71 ± 3.56	<0.001	3.24 ± 1.95	4.41 ± 2.56	0.002

AKI—acute kidney injury; TAVI—transcatheter aortic valve implantation; NR—not relevant; CM—contrast media; SCr—serum creatinine; CrCl—creatinine clearance, BMI—body mass index, BW—body weight. ^ Calculated without CM module.

**Table 4:** Univariable logistic regression analysis for the probability of developing AKI following TAVI.

		*n* = 210		
		OR	95% CI	*p* Value
eGFR reduction by 1 mL/min/1.73 m^2^		1.036	1.017–1.056	<0.001
eGFR <45 mL/min/1.73 m^2^		4.14	1.93–8.9	0.003
Hemoglobin reduction by 1 g/dL		1.33	1.06–1.66	0.013
STS score for mortality		1.0387	1.009–1.069	0.009
Diabetes Mellitus		1.4	0.694–2.835	0.345
Hypertension		1.18	0.381–3.66	0.772
Body mass index		0.974	0.91–1.04	0.458
Gender		0.955	0.471–1.936	0.9
Age		1.041	0.992–1.094	0.1
Left ventricle ejection fraction		0.998	0.965–1.032	0.917
NYHA functional class		1.252	0.474–3.303	0.65
Coronary artery disease		1.836	0.881–3.824	0.105
Peripheral arterial disease		1.818	0.704–4.695	0.217
**Within 30 Days**	CM volume	1.001	0.997–1.003	0.971
	CM/CrCl	1.182	1.058–1.321	0.004
	(CM × Scr)/BMI	1.062	1.014–1.113	0.011
	(CM × Scr)/BW	1.169	1.036–1.139	0.012
	Mehran Score	1.109	1.03–1.195	0.006
	CR4EATME3AD3 score	1.137	1.035–1.249	0.007
**Within 7 Days**	Volume of CM	1.009	0.998–1.004	0.538
	CM/CrCl	1.216	1.073–1.379	0.002
	(CM × Scr)/BMI	1.069	1.012–1.128	0.017
	(CM × Scr)/BW	1.19	1.036–1.367	0.014
	Mehran Score	1.118	1.037–1.205	0.004
	CR4EATME3AD3 score	1.146	1.041–1.262	0.005
**TAVI**	Volume of CM	1.002	0.998–1.007	0.295
	CM/CrCl	1.237	1.069–1.43	0.004
	(CM × SCr)/BMI	1.092	1.021–1.167	<0.001
	(CM × SCr)/BW	1.275	1.069–1.521	0.007
	Mehran Score	1.102	1.013–1.2	0.025
	CR4EATME3AD3 score	1.147	1.043–1.261	0.005
Modified Mehran score ^		1.11	1.028–1.199	0.008
Modified CR4EATME3AD3 score ^		1.146	1.041–1.261	0.005

AKI—acute kidney injury; TAVI—transcatheter aortic valve implantation; eGFR—estimated glomerular filtration rate; NYHA—New-York Heart Association; PAD—peripheral arterial disease, CVD—cardiovascular disease; CM—contrast media; SCr—serum creatinine; CrCl—creatinine clearance; BMI—body mass index, BW—body weight. ^ Calculated without contrast media module.

**Table 5:** Multivariable logistic regression analyses for the prediction of post-TAVI AKI.

				STS Score		Hemoglobin		eGFR ≤ 45 mL/min/1.73 m^2^	
		OR (95% CI)	*p* Value	OR (95% CI)	*p* Value	OR (95% CI)	*p* Value	OR (95% CI)	*p* Value
**Within 30 Days**	Mehran risk score	1.02 (0.93–1.12)	0.63	1.1 (0.94–1.29)	0.24	0.83 (0.63–1.08)	0.165	2.67 (1.1–6.5)	0.03
	CR4EATME3AD3	1.04 (0.92–1.17)	0.514	1.08 (0.92–1.27)	0.355	0.84 (0.65–1.08)	0.168	2.5 (1.01–6.28)	0.049
	CM/CrCl	1.05 (0.9–1.26)	0.539	1.08 (0.91–1.27)	0.345	0.83 (0.64–1.06)	0.137	2.35 (0.85–6.49)	0.099
	(CM× SCr)/BMI	1.02 (0.97–1.08)	0.397	1.09 (0.92–1.29)	0.302	0.84 (0.65–1.08)	0.163	2.48 (0.99–6.19)	0.053
	(CM × SCr)/BW	1.04 (0.91–1.2)	0.548	1.09 (0.92–1.28)	0.328	0.84 (0.65–1.08)	0.163	2.61 (1.04–6.56)	0.041
**Within 7 Days**	Mehran risk score	1.03 (0.94–1.13)	0.518	1.07 (0.91–1.26)	0.41	0.84 (0.65–1.09)	0.194	2.62 (1.07–6.39)	0.034
	CR4EATME3AD3	1.05 (0.93–1.18)	0.417	1.08 (0.92–1.27)	0.364	0.84 (0.65–1.08)	0.17	2.46 (0.98–6.15)	0.054
	CM/CrCl	1.01 (0.94–1.28)	0.216	1.08 (0.91–1.27)	0.364	0.83 (0.64–1.06)	0.123	2.15 (0.8–5.7)	0.125
	(CM × SCr)/BMI	1.02 (0.97–1.09)	0.313	1.09 (0.92–1.28)	0.323	0.83 (0.64–1.07)	0.151	2.44 (1.0–6.1)	0.049
	(CM × SCr)/BW	1.07 (0.92–1.24)	0.377	1.08 (0.92–1.28)	0.361	0.83 (0.64–1.07)	0.15	2.54 (1.03–6.28)	0.044
**TAVI**	Mehran risk score	1.02 (0.92–1.12)	0.761	1.1 (0.94–1.29)	0.228	0.83 (0.64–1.08)	0.173	2.76 (1.11–6.82)	0.028
	CR4EATME3AD3	1.05 (0.93–1.18)	0.429	1.08 (0.92–1.26)	0.357	0.84 (0.65–1.08)	0.175	2.46 (0.98–6.19)	0.054
	CM/CrCl	1.08 (0.89–1.31)	0.43	1.09 (0.92–1.28)	0.309	0.83 (0.64–1.06)	0.134	2.39 (0.85–6.7)	0.1
	(CM × SCr)/BMI	1.03 (0.97–1.12)	0.406	1.1 (0.93–1.29)	0.262	0.83 (0.65–1.07)	0.156	2.48 (0.93–6.45)	0.07
	(CM × SCr)/BW	1.09 (0.88–1.35)	0.414	1.09 (0.93–1.29)	0.287	0.83 (0.64–1.07)	0.159	2.47 (0.94–6.55)	0.067
**Modif-ied ^**	Mehran risk score	1.00 (0.91–1.11)	0.888	1.08 (0.92–1.27)	0.373	0.83 (0.64–1.08)	0.161	2.8 (1.13–6.93)	0.026
	CR4EATME3AD3	1.03 (0.91–1.17)	0.62	1.08 (0.92–1.26)	0.362	0.83 (0.65–1.08)	0.163	2.55 (1.0–6.11)	0.051

TAVI—transcatheter aortic valve implantation; AKI—acute kidney injury; CM—contrast media; CrCl—creatinine clearance; SCr—serum creatinine; BMI—body mass index; BW—body weight. ^ Calculated without contrast media module.

## Data Availability

The data presented in this study was obtained from PMC’s local TAVI prospective registry and is available on request from the corresponding author.
